# 2-methylacetoacetyl-coenzyme A thiolase (beta-ketothiolase) deficiency: one disease - two pathways

**DOI:** 10.1186/s13023-020-01357-0

**Published:** 2020-04-28

**Authors:** Sarah C. Grünert, Jörn Oliver Sass

**Affiliations:** 1 Department of General Pediatrics, Adolescent Medicine and Neonatology, Medical Center – University of Freiburg, Faculty of Medicine, Mathildenstr. 1, 79106 Freiburg, Germany; 2 Research Group Inborn Errors of Metabolism, Department of Natural Sciences & Institute for Functional Gene Analytics (IFGA), Bonn-Rhein-Sieg University of Applied Sciences, von-Liebig-Str. 20, 53359 Rheinbach, Germany

**Keywords:** Ketolysis, Beta-ketothiolase, Organic aciduria, Isoleucine, Ketone body, Metabolic acidosis, Metabolic decompensation, *ACAT1*, Inborn error of metabolism

## Abstract

**Background:**

2-methylacetoacetyl-coenzyme A thiolase deficiency (MATD; deficiency of mitochondrial acetoacetyl-coenzyme A thiolase T2/ “beta-ketothiolase”) is an autosomal recessive disorder of ketone body utilization and isoleucine degradation due to mutations in *ACAT1*.

**Methods:**

We performed a systematic literature search for all available clinical descriptions of patients with MATD. Two hundred forty-four patients were identified and included in this analysis. Clinical course and biochemical data are presented and discussed.

**Results:**

For 89.6% of patients at least one acute metabolic decompensation was reported. Age at first symptoms ranged from 2 days to 8 years (median 12 months). More than 82% of patients presented in the first 2 years of life, while manifestation in the neonatal period was the exception (3.4%). 77.0% (157 of 204 patients) of patients showed normal psychomotor development without neurologic abnormalities.

**Conclusion:**

This comprehensive data analysis provides a systematic overview on all cases with MATD identified in the literature. It demonstrates that MATD is a rather benign disorder with often favourable outcome, when compared with many other organic acidurias.

## Background

**Fig. 1:**
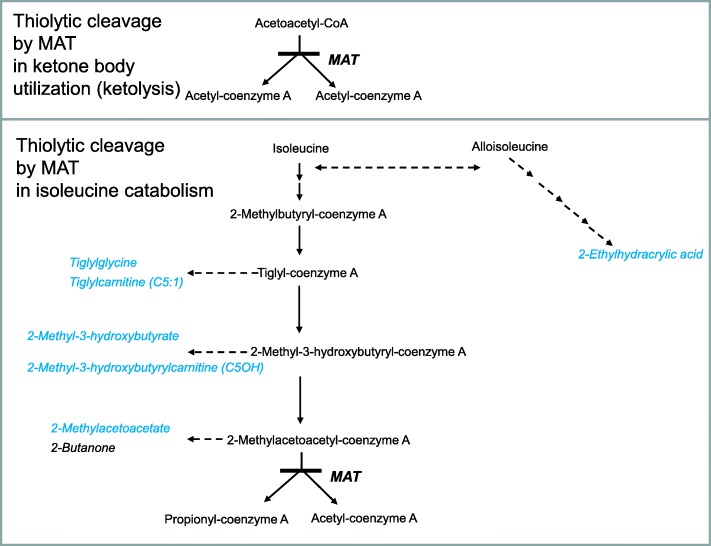
2-methylacetoacetyl-coenzyme A thiolase (MAT) is involved in both ketone body metabolism (top) and isoleucine catabolism (bottom). Metabolites which accumulate in MAT deficiency and can contribute to the diagnosis via abnormalities in urinary organic acids and blood acylcarnitines are printed in light blue

The mitochondrial enzyme 2-methylacetoacetyl-coenzyme A thiolase (MAT; mitochondrial acetoacetyl-coenzyme A thiolase T2/ “beta-ketothiolase”; EC 2.3.1.9) does not only act in ketone body utilization (ketolysis) by catalyzing thiolytic cleavage of acetoacetyl-coenzyme A, but also catalyzes conversion of methylacetoacetyl-coenzyme A in isoleucine catabolism [1] (Fig. 1). Ketone bodies are an important source of energy for extrahepatic organs, in particular for the brain, in times of insufficient energy supply. Consequently, episodes of ketoacidosis with accumulation of isoleucine metabolites (detectable in urinary organic acids and blood acylcarnitines) with or without hypoglycemia are important findings in MAT deficiency (MATD; MIM 203750). Confirmatory testing is available by enzyme activity assays in patient cells (fibroblasts and – in a less reliable manner - lymphocytes) and by mutation analysis in *ACAT1*.

The number of individuals with confirmed MATD has been estimated to be approximately 250 world-wide [1]. In addition to earlier case reports and case series, four recent retrospective studies have investigated MATD patients of various ethnic backgrounds. In 2017, Paquay et al. described 26 French patients born between 1986 and 2014 [2], and Nguyen et al. 41 patients born in Vietnam between 2002 and 2016 [3]. In the same year 2017, Abdelkreem et al. reported 10 MATD patients from Southern India [4] and Grünert et al. 32 patients who were mainly of European/Turkish origin [5]. In 2019, Abdelkreem et al. have provided an update on *ACAT1* variants and their molecular consequences [6]. This has prompted us to perform a systematic assessment of all patients with MATD who have been described in the literature so far, focusing on clinical course and outcome.

## Methods

A systematic literature search was performed in PubMed using the terms “beta-ketothiolase deficiency”, “β-ketothiolase deficiency”, “MAT deficiency”, “2-methylacetoacetyl-coenzyme A thiolase deficiency” and “mitochondrial methylacetoacetyl-coenzyme A thiolase deficiency” in order to collect data on all patients published in the literature to date. The search was performed in November 2019. Additional cases were also included from non-PubMed indexed sources known to the authors, including book chapters. All cases with metabolically, enzymatically and/or genetically documented MATD were included in this study, if relevant clinical information was available from the respective publications.

Following this approach we identified a total of 244 MATD patients. All cases were evaluated and analysed with a special focus on the patients’ age at disease onset, number of metabolic decompensations, clinical course including neurological outcome, treatment, residual enzyme activity and mutations in the *ACAT1* gene. A list of publications included in this paper is displayed in Supplemental Table 1.

Accuracy of age data ranged from hours to years in the different reports. For the calculation of mean and median ages years were converted to months which might lead to an underestimation (i.e. 6 years = 72 months, although the patient might actually have been 6 years and 11 months old).

## Results

Two hundred forty-four cases of MATD with relevant clinical information were identified. An overview on this cohort is given in Table 1. One hundred thirty-two patients were male, 107 female, and the sex of the remaining 5 patients was not reported. The patient cohort included 32 pairs of siblings, including 4 pairs of twins, and two sets of 3 siblings. In one family, father and son were affected. Median age of patients at time of report was 6.0 years (range: neonatal age to 36 years, *n* = 198). 214 (91.1%, *n* = 235) patients were alive at the time of report, while 21 (8.9%) patients had died, most of them from their first metabolic decompensation. Age at death ranged from 7 months to 8 years (median = 16 months).

**Table 1:** Clinical information on 244 patients with MATD reported in the literature

Sex	female *n* = 107
	male *n* = 132
	not reported *n* = 5
Median age at report	6.0 years (neonatal to 36 years, *n* = 198)
Parental consanguinity	33.3% (61/183)
Deceased patients	8.9% (21/235)
Median age at disease onset	12 months (2 days to 8 years, *n* = 205)
Patients with at least 1 metabolic decompensation	89.6% (198/221)
Patients alive with normal development and no neurologic symptoms	77.0% (157/204)
Patients with developmental delay	19.6% (40/204)

Information on parental consanguinity was available for 183 patients. In one third (61/183) consanguinity was reported with most parents being first cousins. In 122 cases parents were reported to not be related. Of the 213 patients of whom the ethnic or geographic background was known, 36.6% were Caucasian, 20.2% of Vietnamese (mainly Kinh), 13.1% of Turkish, 6.1% of Indian (mainly from Hyderabad), 5.6% of Chinese, 3.8% of Japanese and 2.8% of Tunisian ancestry. All other origins accounted for 3 or less patients each.

**Fig. 2:**
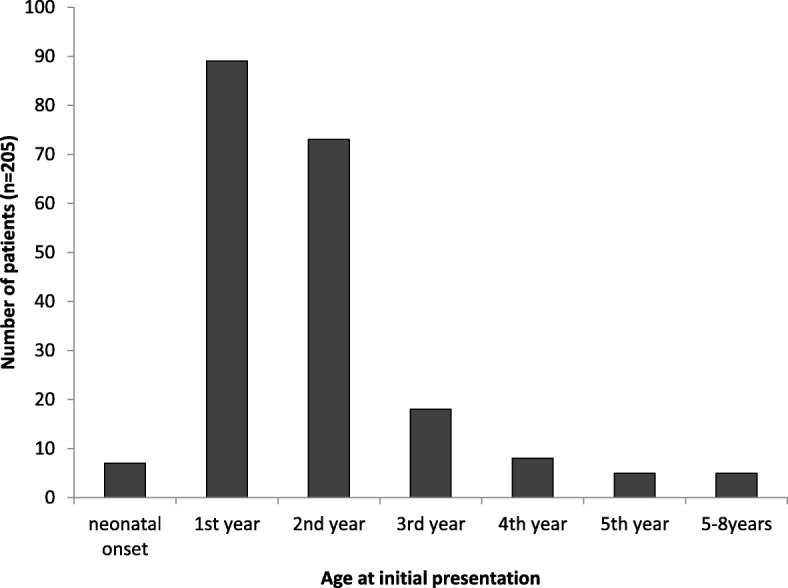
Age at first presentation of 205 patients with MATD and acute symptoms. The vast majority of patients presented within the first 2 years of life while neonatal manifestation was the exception. The latest manifestation was observed at 8 years

Of 205 symptomatic patients age at presentation was reported. Age at first symptoms ranged from 2 days to 8 years (median 12 months). Neonatal presentation (within the first 4 weeks of life) was the exception (*n* = 7/205; 3.4%). 89 (43.4%) and 73 patients (35.6%) presented beyond the neonatal period in the first and second year of life, respectively. All patients manifested within the first decade, the latest manifestation was reported in an 8-year-old patient [2]. An overview on the age at presentation is given in Fig. 2.

**Fig. 3:**
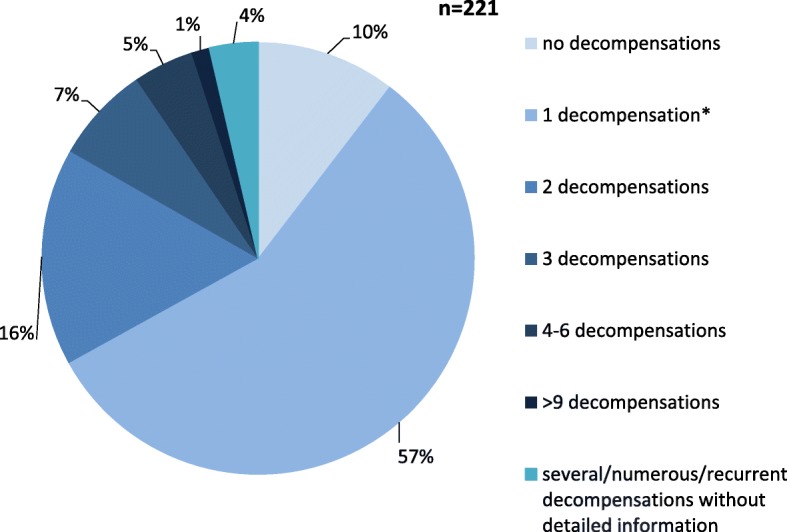
Number of acute decompensations in 221 MATD patients. Around 90% of patients experienced at least one acute metabolic crisis. Patients without acute decompensations were mostly identified by newborn screening or family screening. *This category comprises patients with exactly one decompensation and those with at least one decompensation if no further details were given

The vast majority of patients presented with acute metabolic decompensations, while only 4 children first displayed chronic neurologic symptoms such as muscular hypotonia, seizures, hyporeflexia or impaired motor skills [7–10]. Twenty-eight patients were diagnosed while asymptomatic, either by newborn screening or by family screening due to an affected sibling. Despite the presymptomatic diagnosis, three patients developed a metabolic crisis later during infancy or childhood [5]. Information on the number of metabolic decompensations was available for 221 patients (Fig. 3). Thereof, 198 patients (89.6%) had at least one metabolic crisis, only 73 (33.0%) suffered more than one acute decompensation. Thirty-six patients (16.3%) had 2 decompensations, 26 (11.8%) experienced 3 to 6 decompensations, and in 3 patients (1.4%) more than 9 acute episodes were reported. Twenty-three patients (10.4%) had remained asymptomatic until the age at report.

Acute decompensations were typically characterized by severe metabolic acidosis (pH < 7.0), ketosis and impaired vigilance/coma. Six patients displayed neurologic symptoms compatible with metabolic stroke. Other laboratory findings included hypo- or hyperglycemia, mild hyperammonemia and elevated activities of transaminases. In some patients secondary carnitine deficiency was detected. Interestingly, one patient who developed her first hypoglycemic crisis without ketosis during her neonatal period and had recurrent nonketotic decompensations during infancy and childhood was found to be severely carnitine deficient [7]. The authors hypothesized that the lack of ketosis might have been caused by suppressed beta-oxidation due to carnitine deficiency. Many patients required intensive care treatment including mechanical ventilation. Hemodialysis or peritoneal dialysis was performed in at least 11 cases. In one individual a long-QT interval was noted during the first acute episode [8]. One other patient suffered a cardiac arrest but could be successfully resuscitated [8].

**Fig. 4:**
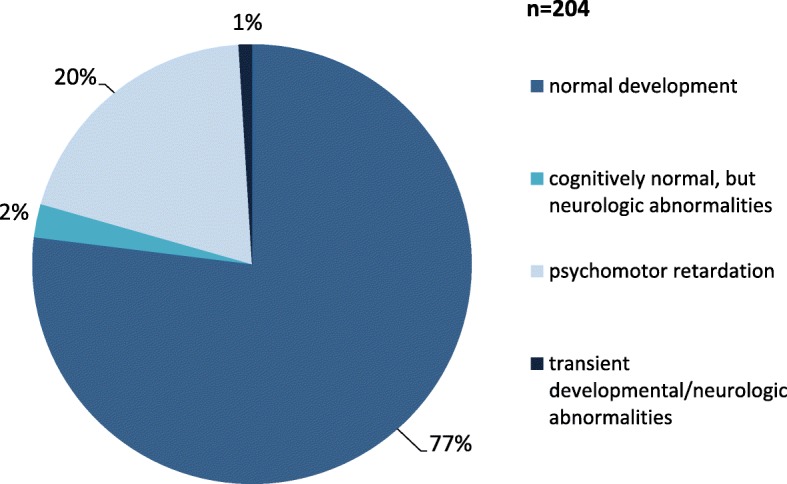
Cognitive development and neurologic complications in 204 MATD patients. 77% of patients showed normal development, while some kind of psychomotor impairment was reported in 20% of patients. 2% of patients were described as cognitively normal, but displayed neurologic symptoms

Information on the neurologic outcome was given for 204 of the 223 surviving patients (Fig. 4). Thereof, 157 (157/204; 77.0%) showed normal psychomotor development without neurologic abnormalities. Further 5 patients (5/204; 2.5%) were reported to be cognitively normal, but displayed neurologic abnormalities including ataxia, hypotonia, choreoathetosis, dystonia and nystagmus. One patient initially showed hypotonia and gross motor developmental delay at the age of 6 months, but later normalized under therapy [9]. One other patient, who displayed ataxia and diplegia at 4 years after adenoidectomy and mumps, had a normal neurological examination and normal intelligence at 7 years [10]. Forty patients (40/204; 19.6%) showed developmental delay, often combined with neurologic symptoms. Only few patients were described as severely disabled, and in most patients with developmental delay mainly the motor development was impaired. In 13 patients (13/204; 6.3%) movement disorders were reported, including dyskinesia, dystonia, choreoathetosis, dysarthria and myoclonic jerks. Seizures were rare in this cohort and only reported in 2 (surviving) patients apart from metabolic decompensations [10, 11].

For 10 of the 21 deceased patients data on neurocognitive development and neurological symptoms are available. Three patients showed developmental delay eventually combined with ataxia, seizures and muscular hypotonia, while seven were reported with normal development until death.

Imaging data (MRI or CT) were available of 71 patients. Brain imaging yielded normal results in 52.1% of patients (37/71) and showed abnormalities in 47.9% (34/71). The most common findings were basal ganglia changes with characteristic T2 hyperintensities affecting mainly the globi pallidi, the putamen, the lentiform and caudate nuclei, but also the substantia nigra. Other findings comprised internal and external capsule lesions, cortical and subcortical atrophy, involvement of the mesencephalon as well as scattered foci in the periventricular and subcortical white matter.

Information on dietary treatment was given for 163 patients. One hundred thirty-four patients (82.2%) followed a specific diet at least transiently. Thereof, 86 patients (86/163; 52.7%) were on either a low isoleucine or low-protein diet. Protein restriction was mostly only mild with a protein intake up to 2 g/kg/day. In 3 patients a natural aversion against high-protein food resulting in a self-selected protein restriction was mentioned [12–14]. Forty-seven patients (47/163; 28.8%) followed a diet restricted in protein and fat, however, the majority of them (41/47) during metabolic crises only. None of the patients received a special amino acid mixture. One patient who was initially misdiagnosed with glutaric aciduria type 1 was put on a diet low in tryptophan and lysine until the correct diagnosis of MAT deficiency was made [15]. In some cases the diet was relaxed during the clinical course. Twenty-nine patients (29/163; 17.8%) never had any dietary restrictions. Avoidance of fasting and a high carbohydrate intake were usually recommended. For 135 patients, data on carnitine treatment was available. Eighty-nine patients (89/135; 65.9%) received oral carnitine supplementation at least transiently (usually between 50 and 200 mg/kg/day), while for 46 patients (46/135; 34.1%) no carnitine supplementation was reported.

Non-neurologic long-term complications seem to be rather rare in this disorder. Some patients showed failure to thrive, which, however, is not unexpected in view of the size of the study cohort. For a single patient with only limited data given, beta-ketothiolase deficiency and fatal cardiomyopathy were purported [16]. Other organ-specific symptoms were not reported. In one female patient a chromosomal abnormality was found in addition to the diagnosis of MATD [17] and most likely is a mere coincidence.

A total of 4 pregnancies in 3 women with MATD have been reported [13, 18, 19]. All were uncomplicated and resulted in healthy offspring. In one mother, Caesarean section was necessary due to fetal bradycardia.

Enzyme activity studies were performed in 105 patients. They consistently confirmed decreased or absent MAT activity in fibroblasts, lymphocytes or EBV-transformed lymphoblastoid cells.

Results of *ACAT1* mutation analysis were reported for 164 of the 244 patients. Eighty-five patients (85/164; 51.8%) were found to be homozygous for *ACAT1* mutations, and in 78 patients (78/164; 47.6%) compound heterozygosity was observed. In one case no details were given, but the disorder was described as “genetically proven”, and in another patient, only one mutation could be identified. Notably, only for 29 of the 67 homozygous patients for whom information on parental consanguinity was available, parents were actually reported to be related. The most common mutation was the *ACAT1* c.622C > T, p.(Arg208*) variant, a stop mutation that was found in at least 36 individuals (50% in homozygosity and 50% in compound heterozygosity) of Vietnamese, Dutch and Turkish origin [3, 5]. Thus, it may be a frequent cause of MATD in a wider range of populations. Two other variants that were identified in 12 and 10 patients, respectively, were the splice site variant c.1006-1G > C and the missense mutation c.949G > A, p.(AspD317Asn), that is located at an exonic splice enhancer site and results in exon 10 skipping [20]. All other mutations were described in less than 10 individuals.

## Discussion

This reports aims at a comprehensive overview on the clinical course and outcome of all patients with MATD published in the literature so far. Our analysis confirms that MATD is a panethnic disease.

The vast majority of patients manifested acutely with a metabolic decompensation. 8.9% of patients had died, many of them during their first metabolic crisis, when the diagnosis was still unknown. This suggests that awareness of this metabolic disorder may be a key issue for avoiding fatal sequences. Consequently, MATD may be suitable as a target disease of newborn screening programs, in particular in view of the fact that only few patients (2.5%) present with symptoms within the first 4 weeks of life. However, as C5:1 and C5OH acylcarnitine levels in blood may even be normal during acute crises [18], current approaches to newborn screening do not necessarily identify individuals at risk [22].

In patients in whom the diagnosis of MATD was based on metabolite data only (without rather labile 2-methylacetoacetic acid), but not confirmed on enzyme and/or mutation level, it needs to be considered that patients were possibly not affected by MATD but by HSD10 mitochondrial disease (HSD10MD; OMIM 300438). HSD10 mitochondrial disease is caused by a hemizygous or heterozygous mutation in the *HSD17B10* gene [23, 24]. The suspicion that patients actually had HSD10 mitochondrial disease prompted us to exclude two brothers from this study, who have been reported as beta-ketothiolase deficient by Jänisch et al. 1993 and Hesse et al. 2004 [25, 26], but in whom we consider published clinical and enzyme data rather nonsuggestive for MATD.

The overall neurologic outcome of MATD patients is rather favorable with more than 75% showing normal development without neurologic symptoms. Neurological impairment can occur 1) pre-existing to the first metabolic ketoacidosis, 2) as a consequence of (a severe) ketoacidotic episode (stroke-like picture) or 3) independent of ketoacidotic episodes [21]. The most common imaging findings were basal ganglia (striatal) injuries. In agreement with our recent overview on an inherited deficiency of ketogenesis, 3-hydroxy-3-methylglutaryl-coenzyme A dehydrogenase deficiency [27], we observed no consistent association between imaging findings and clinical features in MATD. Not all patients exhibiting striatal injuries were neurologically symptomatic, and in others with clear neurologic symptoms the MRI was described as normal.

The detailedness of the clinical data given in different case reports varied broadly. Especially the follow-up periods of patients were short in some reports. Therefore, data for frequencies of ketoacidotic crises, neurologic outcome and prognosis have to be interpreted with appropriate caution.

Although most patients were recommended a specific diet with some restriction of isoleucine/protein and avoidance of fat excess, it can be assumed that the avoidance of fasting is probably the main factor to prevent metabolic decompensations. For none of the patients supplementation of a special amino acid mixture was reported after diagnosis with MATD, however, the majority of patients was supplemented with L-carnitine at least temporarily.

It is known that the genotype and clinical phenotype do not correlate in MATD [5, 6]. Furthermore, MATD patients may show variable clinical phenotypes even in case of common genotype and ancestry, as well as similar personal histories and daily life environments [3].

## Conclusion

Compared with many other organic acid disorders, MATD often presents with a rather positive outcome, if fatal risks which are in particular associated with the first metabolic decompensation, are managed appropriately.

## Supplementary information

**Additional file 1: Table S1.** Publications included in this literature review for the analysis of clinical, biochemical and genetic data.

## Data Availability

Raw data of this analysis are available on request.

## Abbreviation

MATD: 2-methylacetoacetyl-coenzyme A thiolase (beta-ketothiolase) deficiency

## References

[1] Sass JO, Fukao T, Mitchell GA (2018). Inborn errors of ketone body metabolism and transport: an update for the clinic and for clinical laboratories. J Inborn Errors Metab Screen.

[2] Paquay S, Bourillon A, Pichard S, Benoist J-F, de Lonlay P, Dobbelaere D (2017). Mitochondrial acetoacetyl-CoA thiolase deficiency: basal ganglia impairment may occur independently of ketoacidosis. J Inherit Metab Dis.

[3] Nguyen KN, Abdelkreem E, Colombo R, Hasegawa Y, Can NTB, Bui TP (2017). Characterization and outcome of 41 patients with beta-ketothiolase deficiency: 10 years’ experience of a medical center in northern Vietnam. J Inherit Metab Dis.

[4] Abdelkreem E, Akella RRD, Dave U, Sane S, Otsuka H, Sasai H (2017). Clinical and mutational characterizations of ten Indian patients with Beta-Ketothiolase deficiency. JIMD Rep.

[5] Grünert SC, Schmitt RN, Schlatter SM, Gemperle-Britschgi C, Balcı MC, Berg V (2017). Clinical presentation and outcome in a series of 32 patients with 2-methylacetoacetyl-coenzyme a thiolase (MAT) deficiency. Mol Genet Metab.

[6] Abdelkreem E, Harijan RK, Yamaguchi S, Wierenga RK, Fukao T (2019). Mutation update on ACAT1 variants associated with mitochondrial acetoacetyl-CoA thiolase (T2) deficiency. Hum Mutat.

[7] Alijanpour M, Sasai H, Abdelkreem E, Ago Y, Soleimani S, Moslemi L (2019). Beta-ketothiolase deficiency: a case with unusual presentation of nonketotic hypoglycemic episodes due to coexistent probable secondary carnitine deficiency. JIMD Rep.

[8] Daum RS, Scriver CR, Mamer OA, Delvin E, Lamm P, Goldman H (1973). An inherited disorder of isoleucine catabolism causing accumulation of alpha-methylacetoacetate and alpha-methyl-beta -hydroxybutyrate, and intermittent metabolic acidosis. Pediatr Res.

[9] Zhang G, Fukao T, Sakurai S, Yamada K, Gibson KM, Kondo N. Identification of Alu-mediated, large deletion-spanning exons 2-4 in a patient with mitochondrial acetoacetyl-CoA thiolase deficiency. Mol Genet Metab. 2006;89:222–6. 10.1016/j.ymgme.2006.06.010 16935016

[10] Ozand PT, Rashed M, Gascon GG, al Odaib A, Shums A, Nester M (1994). 3-Ketothiolase deficiency: a review and four new patients with neurologic symptoms. Brain and Development.

[11] Abdelkreem E, Akella RRD, Dave U, Sane S, Otsuka H, Sasai H, et al. Clinical and Mutational Characterizations of Ten Indian Patients with Beta-Ketothiolase Deficiency. JIMD Rep. 2017;35:59–65. 10.1007/8904_2016_26 PMC5585108 27928777

[12] de Kremer RD, de Boldini CD, Kelley RI, Civallero GE (1997). Mitochondrial 2-methylacetoacetyl-CoA thiolase deficiency in Argentina. Medicina (B Aires).

[13] Sewell AC, Herwig J, Wiegratz I, Lehnert W, Niederhoff H, Song XQ (1998). Mitochondrial acetoacetyl-CoA thiolase (beta-ketothiolase) deficiency and pregnancy. J Inherit Metab Dis.

[14] Sabetta G, Bachmann C, Giardini O, Castro M, Gambarara M, Vici CD (1987). Beta-Ketothiolase deficiency with favourable evolution. J Inherit Metab Dis.

[15] Wojcik MH, Wierenga KJ, Rodan LH, Sahai I, Ferdinandusse S, Genetti CA (2018). Beta-Ketothiolase deficiency presenting with metabolic stroke after a Normal newborn screen in two individuals. JIMD Rep.

[16] Henry CG, Strauss AW, Keating JP, Hillman RE (1981). Congestive cardiomyopathy associated with beta-ketothiolase deficiency. J Pediatr.

[17] Gray RG, Lowther GW, Littlewood JM, Middleton B, Bennett MJ (1984). A case of 2-methylacetoacetyl CoA thiolase deficiency with coincidental chromosome abnormalities. J Med Genet.

[18] Fukao T, Maruyama S, Ohura T, Hasegawa Y, Toyoshima M, Haapalainen AM (2012). Three Japanese Patients with Beta-Ketothiolase Deficiency Who Share a Mutation, c.431A>C (H144P) in ACAT1 : Subtle Abnormality in Urinary Organic Acid Analysis and Blood Acylcarnitine Analysis Using Tandem Mass Spectrometry. JIMD Rep.

[19] Kayani R, Botros S, Moore P (2013). Beta-ketothiolase deficiency and pregnancy. Int J Obstet Anesth.

[20] Otsuka H, Sasai H, Nakama M, Aoyama Y, Abdelkreem E, Ohnishi H (2016). Exon 10 skipping in ACAT1 caused by a novel c.949G>A mutation located at an exonic splice enhancer site. Mol Med Rep.

[21] Fukao T, Sasai H, Aoyama Y, Otsuka H, Ago Y, Matsumoto H (2019). Recent advances in understanding beta-ketothiolase (mitochondrial acetoacetyl-CoA thiolase, T2) deficiency. J Hum Genet.

[22] Sarafoglou K, Matern D, Redlinger-Grosse K, Bentler K, Gaviglio A, Harding CO, Rinaldo P. Siblings With Mitochondrial Acetoacetyl-CoA Thiolase Deficiency Not Identified by Newborn Screening. Pediatrics 128:e246-50. 10.1542/peds.2010-3918 21669895

[23] Ofman R, Ruiter JP, Feenstra M, Duran M, Poll-The BT, Zschocke J, Ensenauer R, Lehnert W, Sass JO, Sperl W, Wanders RJ. 2-Methyl-3-hydroxybutyryl-CoA dehydrogenase deficiency is caused by mutations in the HADH2 gene. Am J Hum Genet. 2003;72:1300-7. 10.1086/375116 PMC1180283 12696021

[24] Oerum S, Roovers M, Leichsenring M, Acquaviva-Bourdain C, Beermann F, Gemperle-Britschgi C, Fouilhoux A, Korwitz-Reichelt A, Bailey HJ, Droogmans L, Oppermann U, Sass JO, Yue WW. Novel patient missense mutations in the HSD17B10 gene affect dehydrogenase and mitochondrial tRNA modification functions of the encoded protein. Biochim Biophys Acta Mol Basis Dis. 2017;1863:3294-3302. 10.1016/j.bbadis.2017.09.002 28888424

[25] Jänisch W, Hesse V, Fiedler B, Förster H, Böhles H. Pathomorpholigical findings in ketothiolase deficiency. Zentralbl Pathol. 1993;139:245-53. 8218125

[26] Hesse V, Sewell AC, Böhles H, Haberland H, Middleton B, Fiedler B, Förster H, Janisch W. Cardiomyopathy in β-ketothiolase deficiency. In: Metabolic Cardiomyopathy. Böhles H, Sewell AC, editors. Stuttgart: medpharm, 2004. p 35-42.

[27] Grünert SC, Sass JO. 3-hydroxy-3-methylglutaryl-coenzyme A lyase deficiency: one disease - many faces. Orphanet J Rare Dis. 2020;15:48. 10.1186/s13023-020-1319-7 PMC7023732 32059735

