# Supplementary material for: 2-methylacetoacetyl-coenzyme A thiolase (beta-ketothiolase) deficiency: one disease - two pathways
Source: Orphanet J Rare Dis. 2020 Apr 28;15:106. doi: 10.1186/s13023-020-01357-0 (PMC7187484; doi:10.1186/s13023-020-01357-0)
Supplement: Supplementary file 1 — Additional file 1: Table S1. Publications included in this literature review for the analysis of clinical, biochemical and genetic data. [file 13023_2020_1357_MOESM1_ESM.docx]

**Supplemental Table 1: Publications included in this literature review for the analysis of clinical, biochemical and genetic data.**

1. Abdelkreem E, Akella RRD, Dave U, Sane S, Otsuka H, Sasai H, et al. Clinical and Mutational Characterizations of Ten Indian Patients with Beta-Ketothiolase Deficiency. JIMD Rep. 2017;35:59–65.

2. Akella RRD, Aoyama Y, Mori C, Lingappa L, Cariappa R, Fukao T. Metabolic encephalopathy in beta-ketothiolase deficiency: the first report from India. Brain Dev. 2014;36:537–40.

3. Alijanpour M, Sasai H, Abdelkreem E, Ago Y, Soleimani S, Moslemi L, et al. Beta-ketothiolase deficiency: A case with unusual presentation of nonketotic hypoglycemic episodes due to coexistent probable secondary carnitine deficiency. JIMD Rep. 2019;46:23–7.

4. Altintaş B, Teziç T, Coşkun T, Ozalp I, Kükner S, Kaya A. Beta-ketothiolase deficiency. A case report. Turk J Pediatr. 1992;34:43–6.

5. Aramaki S, Lehotay D, Sweetman L, Nyhan WL, Winter SC, Middleton B. Urinary excretion of 2-methylacetoacetate, 2-methyl-3-hydroxybutyrate and tiglylglycine after isoleucine loading in the diagnosis of 2-methylacetoacetyl-CoA thiolase deficiency. J Inherit Metab Dis. 1991;14:63–74.

6. Arica V, Arica SG, Dag H, Onur H, Obut O, Gülbayzar S. Beta-ketothiolase deficiency brought with lethargy: case report. Hum Exp Toxicol. 2011;30:1724–7.

7. Bennett MJ, Littlewood JM, MacDonald A, Pollitt RJ, Thompson J. A case of beta-ketothiolase deficiency. J Inherit Metab Dis. 1983;6:157.

8. Brismar J, Ozand PT. CT and MR of the brain in the diagnosis of organic acidemias. Experiences from 107 patients. Brain Dev. 1994;16 Suppl:104–24.

9. Buhaş D, Bernard G, Fukao T, Décarie J-C, Chouinard S, Mitchell GA. A treatable new cause of chorea: beta-ketothiolase deficiency. Mov Disord. 2013;28:1054–6.

10. Canda, Ebru, Yazici, Havva, Kalkan Uca, Sema, Gemperle-Britschgi, Corinne, Habif, Sara, Onay, Hüseyin, et al. Recurrent ketoacidosis: Is it a ketone metabolism disorder? İzmir Dr Behçet Uz Çocuk Hast Dergisi. 2018;8:151–121.

11. Cardoso, Francisco. Commentary on the article by Buhas et al 2013. Movement Disorders. 2013;28:1056.

12. Catanzano F, Ombrone D, Di Stefano C, Rossi A, Nosari N, Scolamiero E, et al. The first case of mitochondrial acetoacetyl-CoA thiolase deficiency identified by expanded newborn metabolic screening in Italy: the importance of an integrated diagnostic approach. J Inherit Metab Dis. 2010;33 Suppl 3:S91-94.

13. Cromby CH, Manning NJ, Pollitt RJ, Powell S, Bennett MJ. 6-Methyluracil excretion in 2-methylacetoacetyl-CoA thiolase deficiency and in two children with an unexplained recurrent ketoacidaemia. J Inherit Metab Dis. 1994;17:81–4.

14. Cubillo Serna I, Suárez Fernández J, Merino Arribas JM, Díaz Ruiz J, Bustamante Hervás C, De Frutos Martínez C. [Mitochondrial acetoacetyl-CoA thiolase deficiency: neonatal onset]. An Pediatr (Barc). 2007;67:381–4.

15. Daum RS, Lamm PH, Mamer OA, Scriver CR. A “new” disorder of isoleucine catabolism. Lancet. 1971;2:1289–90.

16. Daum RS, Scriver CR, Mamer OA, Delvin E, Lamm P, Goldman H. An inherited disorder of isoleucine catabolism causing accumulation of alpha-methylacetoacetate and alpha-methyl-beta -hydroxybutyrate, and intermittent metabolic acidosis. Pediatr Res. 1973;7:149–60.

17. de Kremer RD, de Boldini CD, Kelley RI, Civallero GE. [Mitochondrial 2-methylacetoacetyl-CoA thiolase deficiency in Argentina]. Medicina (B Aires). 1997;57:52–8.

18. Elleau C, Parrot-Roulaud F, Perel Y, Divry P, Rolland MO, Zabot MT, et al. [Beta-ketothiolase deficiency: a case of ketoacidosis with hyperglycinemia]. Pediatrie. 1992;47:185–9.

19. Estrella J, Wilcken B, Carpenter K, Bhattacharya K, Tchan M, Wiley V. Expanded newborn screening in New South Wales: missed cases. J Inherit Metab Dis. 2014;37:881–7.

20. Fontaine M, Briand G, Ser N, Armelin I, Rolland MO, Degand P, et al. Metabolic studies in twin brothers with 2-methylacetoacetyl-CoA thiolase deficiency. Clin Chim Acta. 1996;255:67–83.

21. Frazier DM, Millington DS, McCandless SE, Koeberl DD, Weavil SD, Chaing SH, et al. The tandem mass spectrometry newborn screening experience in North Carolina: 1997-2005. J Inherit Metab Dis. 2006;29:76–85.

22. Fukao T, Yamaguchi S, Wakazono A, Orii T, Hoganson G, Hashimoto T. Identification of a novel exonic mutation at -13 from 5’ splice site causing exon skipping in a girl with mitochondrial acetoacetyl-coenzyme A thiolase deficiency. J Clin Invest. 1994;93:1035–41.

23. Fukao T, Song XQ, Yamaguchi S, Orii T, Wanders RJ, Poll-The BT, et al. Mitochondrial acetoacetyl-coenzyme A thiolase gene: a novel 68-bp deletion involving 3’ splice site of intron 7, causing exon 8 skipping in a Caucasian patient with beta-ketothiolase deficiency. Hum Mutat. 1995;5:94–6.

24. Fukao T, Kodama A, Aoyanagi N, Tsukino R, Uemura S, Song XQ, et al. Mild form of beta-ketothiolase deficiency (mitochondrial acetoacetyl-CoA thiolase deficiency) in two Japanese siblings: identification of detectable residual activity and cross-reactive material in EB-transformed lymphocytes. Clin Genet. 1996;50:263–6.

25. Fukao T, Boneh A, Aoki Y, Kondo N. A novel single-base substitution (c.1124A>G) that activates a 5-base upstream cryptic splice donor site within exon 11 in the human mitochondrial acetoacetyl-CoA thiolase gene. Mol Genet Metab. 2008;94:417–21.

26. Fukao T, Scriver CR, Kondo N, t2 Collaborative Working Group. The clinical phenotype and outcome of mitochondrial acetoacetyl-CoA thiolase deficiency (beta-ketothiolase or T2 deficiency) in 26 enzymatically proved and mutation-defined patients. Mol Genet Metab. 2001;72:109–14.

27. Fukao T, Nakamura H, Nakamura K, Perez-Cerda C, Baldellou A, Barrionuevo CR, et al. Characterization of six mutations in five Spanish patients with mitochondrial acetoacetyl-CoA thiolase deficiency: effects of amino acid substitutions on tertiary structure. Mol Genet Metab. 2002;75:235–43.

28. Fukao T, Zhang G, Rolland M-O, Zabot M-T, Guffon N, Aoki Y, et al. Identification of an Alu-mediated tandem duplication of exons 8 and 9 in a patient with mitochondrial acetoacetyl-CoA thiolase (T2) deficiency. Mol Genet Metab. 2007;92:375–8.

29. Fukao T, Aoyama Y, Murase K, Hori T, Harijan RK, Wierenga RK, et al. Development of MLPA for human ACAT1 gene and identification of a heterozygous Alu-mediated deletion of exons 3 and 4 in a patient with mitochondrial acetoacetyl-CoA thiolase (T2) deficiency. Mol Genet Metab. 2013;110:184–7.

30. Fukao T, Yamaguchi S, Scriver CR, Dunbar G, Wakazono A, Kano M, et al. Molecular studies of mitochondrial acetoacetyl-coenzyme A thiolase deficiency in the two original families. Hum Mutat. 1993;2:214–20.

31. Fukao T, Yamaguchi S, Orii T, Hashimoto T. Molecular basis of beta-ketothiolase deficiency: mutations and polymorphisms in the human mitochondrial acetoacetyl-coenzyme A thiolase gene. Hum Mutat. 1995;5:113–20.

32. Fukao T, Song XQ, Yamaguchi S, Kondo N, Orii T, Matthieu JM, et al. Identification of three novel frameshift mutations (83delAT, 754insCT, and 435 + 1G to A) of mitochondrial acetoacetyl-coenzyme A thiolase gene in two Swiss patients with CRM-negative beta-ketothiolase deficiency. Hum Mutat. 1997;9:277–9.

33. Fukao T, Maruyama S, Ohura T, Hasegawa Y, Toyoshima M, Haapalainen AM, et al. Three Japanese Patients with Beta-Ketothiolase Deficiency Who Share a Mutation, c.431A>C (H144P) in ACAT1 : Subtle Abnormality in Urinary Organic Acid Analysis and Blood Acylcarnitine Analysis Using Tandem Mass Spectrometry. JIMD Rep. 2012;3:107–15.

34. Fukao T, Matsuo N, Zhang GX, Urasawa R, Kubo T, Kohno Y, et al. Single base substitutions at the initiator codon in the mitochondrial acetoacetyl-CoA thiolase (ACAT1/T2) gene result in production of varying amounts of wild-type T2 polypeptide. Hum Mutat. 2003;21:587–92.

35. Fukao T, Zhang GX, Sakura N, Kubo T, Yamaga H, Hazama A, et al. The mitochondrial acetoacetyl-CoA thiolase (T2) deficiency in Japanese patients: urinary organic acid and blood acylcarnitine profiles under stable conditions have subtle abnormalities in T2-deficient patients with some residual T2 activity. J Inherit Metab Dis. 2003;26:423–31.

36. Fukao T, Horikawa R, Naiki Y, Tanaka T, Takayanagi M, Yamaguchi S, et al. A novel mutation (c.951C>T) in an exonic splicing enhancer results in exon 10 skipping in the human mitochondrial acetoacetyl-CoA thiolase gene. Mol Genet Metab. 2010;100:339–44.

37. Fukao T, Nguyen HT, Nguyen NT, Vu DC, Can NTB, Pham ATV, et al. A common mutation, R208X, identified in Vietnamese patients with mitochondrial acetoacetyl-CoA thiolase (T2) deficiency. Mol Genet Metab. 2010;100:37–41.

38. Galanello R, Cao A, Olivieri N. Induction of fetal hemoglobin in the presence of increased 3-hydroxybutyric acid associated with beta-ketothiolase deficiency. N Engl J Med. 1994;331:746–7.

39. Gibson KM, Elpeleg ON, Bennett MJ. beta-Ketothiolase (2-methylacetoacetyl-coenzyme A thiolase) deficiency: identification of two patients in Israel. J Inherit Metab Dis. 1996;19:698–9.

40. Gibson KM, Feigenbaum AS. Phenotypically mild presentation in a patient with 2-methylacetoacetyl-coenzyme A (beta-keto)thiolase deficiency. J Inherit Metab Dis. 1997;20:712–3.

41. Gray RG, Lowther GW, Littlewood JM, Middleton B, Bennett MJ. A case of 2-methylacetoacetyl CoA thiolase deficiency with coincidental chromosome abnormalities. J Med Genet. 1984;21:397.

42. Halvorsen S, Stokke O, Jellum E. A variant form of 2-methyl-3-hydroxybutyric and 2-methylacetoacetic aciduria. Acta Paediatr Scand. 1979;68:123–8.

43. Henry CG, Strauss AW, Keating JP, Hillman RE. Congestive cardiomyopathy associated with beta-ketothiolase deficiency. J Pediatr. 1981;99:754–7.

44. Hillman RE, Keating JP. Beta-ketothiolase deficiency as a cause of the “ketotic hyperglycinemia syndrome.” Pediatrics. 1974;53:221–5.

45. Hiyama K, Sakura N, Matsumoto T, Kuhara T. Deficient beta-ketothiolase activity in leukocytes from a patient with 2-methylacetoacetic aciduria. Clin Chim Acta. 1986;155:189–94.

46. Kayani R, Botros S, Moore P. Beta-ketothiolase deficiency and pregnancy. Int J Obstet Anesth. 2013;22:260–1.

47. Keating JP, Feigin RD, Tenenbaum SM, Hillman RE. Hyperglycinemia with ketosis due to a defect in isoleucine metabolism: a preliminary report. Pediatrics. 1972;50:890–5.

48. Kılıç-Yıldırım G, Durmuş-Aydoğdu S, Ceylaner S, Sass JO. Beta-ketothiolase deficiency: An unusual cause of recurrent ketoacidosis. Turk J Pediatr. 2017;59:471–4.

49. Köse, Melis Demir, Canda, Ebru, Kagnici, Mehtap, İşgüder, Rana, Ünalp, Aycan, Uçar, Sema Kalkan, et al. Two Siblings with Beta-Ketothiolase Deficiency: One Genetic Defect Two Different Pictures. J Pediatr Res. 2016;3:113–6.

50. Law C-Y, Lam C-W, Ching C, Yau K-CE, Ho T, Lai C, et al. NMR-based urinalysis for beta-ketothiolase deficiency. Clin Chim Acta. 2015;438:222–5.

51. Leonard JV, Middleton B, Seakins JW. Acetoacetyl CoA thiolase deficiency presenting as ketotic hypoglycemia. Pediatr Res. 1987;21:211–3.

52. Merinero, B, Pérez-Cerdá, C, García, M J, Carrasco, S, Lama, R, Ugarte, M, et al. beta-Ketothiolase Deficiency: Two Siblings with Different Clinical Conditions. J Inherit Metab Dis. 1987;Suppl 2:276–8.

53. Middleton B, Gray RG, Bennett MJ. Two cases of beta-ketothiolase deficiency: a comparison. J Inherit Metab Dis. 1984;7 Suppl 2:131–2.

54. Monastiri K, Amri F, Limam K, Kaabachi N, Guediche MN. beta-Ketothiolase (2-methylacetoacetyl-CoA thiolase) deficiency: a frequent disease in Tunisia? J Inherit Metab Dis. 1999;22:932–3.

55. Mrázová L, Fukao T, Hálovd K, Gregová E, Kohút V, Pribyl D, et al. Two novel mutations in mitochondrial acetoacetyl-CoA thiolase deficiency. J Inherit Metab Dis. 2005;28:235–6.

56. Nagasawa H, Yamaguchi S, Orii T, Schutgens RB, Sweetman L, Hashimoto T. Heterogeneity of defects in mitochondrial acetoacetyl-CoA thiolase biosynthesis in fibroblasts from four patients with 3-ketothiolase deficiency. Pediatr Res. 1989;26:145–9.

57. Nakamura K, Fukao T, Perez-Cerda C, Luque C, Song XQ, Naiki Y, et al. A novel single-base substitution (380C>T) that activates a 5-base downstream cryptic splice-acceptor site within exon 5 in almost all transcripts in the human mitochondrial acetoacetyl-CoA thiolase gene. Mol Genet Metab. 2001;72:115–21.

58. Ngu LH, Zabedah MY, Shanti B, Teh SH. Biochemical profiling in two siblings with mitochondrial 2-methylacetoacetyl-CoA thiolase deficiency. Malays J Pathol. 2008;30:109–14.

59. Nguyen KN, Abdelkreem E, Colombo R, Hasegawa Y, Can NTB, Bui TP, et al. Characterization and outcome of 41 patients with beta-ketothiolase deficiency: 10 years’ experience of a medical center in northern Vietnam. J Inherit Metab Dis. 2017;40:395–401.

60. O’Neill ML, Kuo F, Saigal G. MRI of pallidal involvement in Beta-ketothiolase deficiency. J Neuroimaging. 2014;24:414–7.

61. Otsuka H, Sasai H, Nakama M, Aoyama Y, Abdelkreem E, Ohnishi H, et al. Exon 10 skipping in ACAT1 caused by a novel c.949G>A mutation located at an exonic splice enhancer site. Mol Med Rep. 2016;14:4906–10.

62. Ozand PT, Rashed M, Gascon GG, al Odaib A, Shums A, Nester M, et al. 3-Ketothiolase deficiency: a review and four new patients with neurologic symptoms. Brain Dev. 1994;16 Suppl:38–45.

63. Pang CP, Law LK, Mak YT, Shek CC, Cheung KL, Mak TW, et al. Biochemical investigation of young hospitalized Chinese children: results over a 7-year period. Am J Med Genet. 1997;72:417–21.

64. Paquay S, Bourillon A, Pichard S, Benoist J-F, de Lonlay P, Dobbelaere D, et al. Mitochondrial acetoacetyl-CoA thiolase deficiency: basal ganglia impairment may occur independently of ketoacidosis. J Inherit Metab Dis. 2017;40:415–22.

65. Pollitt RJ. The occurrence of substituted 3-methyl-3-hydroxyglutaric acids in urine in propionic acidaemia and in beta-ketothiolase deficiency. Biomed Mass Spectrom. 1983;10:253–7.

66. Rajan D, Constance LSL, Brandon P. Beta-ketothiolase deficiency in a Malaysian infant. Med J Malaysia. 2019;74:174–5.

67. Renom G, Fontaine M, Rolland MO, Duprey J, Degand PM, Dobbelaere D. A new case of 2-methylacetoacetyl-CoA thiolase deficiency? J Inherit Metab Dis. 2000;23:751–3.

68. Riudor E, Ribes A, Perez-Cerda C, Arranz JA, Mora J, Yeste D, et al. Metabolic coma with ketoacidosis and hyperglycaemia in 2-methylacetoacetyl-CoA thiolase deficiency. J Inherit Metab Dis. 1995;18:748–9.

69. Robinson BH, Sherwood WG, Taylor J, Balfe JW, Mamer OA. Acetoacetyl CoA thiolase deficiency: a cause of severe ketoacidosis in infancy simulating salicylism. J Pediatr. 1979;95:228–33.

70. Sabetta G, Bachmann C, Giardini O, Castro M, Gambarara M, Vici CD, et al. beta-Ketothiolase deficiency with favourable evolution. J Inherit Metab Dis. 1987;10:405–6.

71. Sakurai S, Fukao T, Haapalainen AM, Zhang G, Yamada K, Lilliu F, et al. Kinetic and expression analyses of seven novel mutations in mitochondrial acetoacetyl-CoA thiolase (T2): identification of a Km mutant and an analysis of the mutational sites in the structure. Mol Genet Metab. 2007;90:370–8.

72. Sarafoglou K, Matern D, Redlinger-Grosse K, Bentler K, Gaviglio A, Harding CO, et al. Siblings with mitochondrial acetoacetyl-CoA thiolase deficiency not identified by newborn screening. Pediatrics. 2011;128:e246-250.

73. Sasai H, Aoyama Y, Otsuka H, Abdelkreem E, Nakama M, Hori T, et al. Single-nucleotide substitution T to A in the polypyrimidine stretch at the splice acceptor site of intron 9 causes exon 10 skipping in the ACAT1 gene. Mol Genet Genomic Med. 2017;5:177–84.

74. Schutgens RB, Middleton B, vd Blij JF, Oorthuys JW, Veder HA, Vulsma T, et al. Beta-ketothiolase deficiency in a family confirmed by in vitro enzymatic assays in fibroblasts. Eur J Pediatr. 1982;139:39–42.

75. Scolamiero E, Cozzolino C, Albano L, Ansalone A, Caterino M, Corbo G, et al. Targeted metabolomics in the expanded newborn screening for inborn errors of metabolism. Mol Biosyst. 2015;11:1525–35.

76. Sewell AC, Herwig J, Wiegratz I, Lehnert W, Niederhoff H, Song XQ, et al. Mitochondrial acetoacetyl-CoA thiolase (beta-ketothiolase) deficiency and pregnancy. J Inherit Metab Dis. 1998;21:441–2.

77. Shiasi Arani K, Soltani B. First report of 3-oxothiolase deficiency in iran. Int J Endocrinol Metab. 2014;12:e10960.

78. Søvik O. Mitochondrial 2-methylacetoacetyl-CoA thiolase deficiency: an inborn error of isoleucine and ketone body metabolism. J Inherit Metab Dis. 1993;16:46–54.

79. Su L, Li X, Lin R, Sheng H, Feng Z, Liu L. Clinical and molecular analysis of 6 Chinese patients with isoleucine metabolism defects: identification of 3 novel mutations in the HSD17B10 and ACAT1 gene. Metab Brain Dis. 2017;32:2063–71.

80. Sundaram S, Nair M, Namboodhiri S, Menon RN. Mitochondrial acetoacetyl-CoA thiolase enzyme deficiency in a 9-month old boy: Atypical urinary metabolic profile with a novel homozygous mutation in ACAT1 gene. Neurol India. 2018;66:1802–4.

81. Thümmler S, Dupont D, Acquaviva C, Fukao T, de Ricaud D. Different clinical presentation in siblings with mitochondrial acetoacetyl-CoA thiolase deficiency and identification of two novel mutations. Tohoku J Exp Med. 2010;220:27–31.

82. Tilbrook LK, Slater J, Agarwal A, Cyriac J. An unusual cause of interference in a salicylate assay caused by mitochondrial acetoacetyl-CoA thiolase deficiency. Ann Clin Biochem. 2008;45 Pt 5:524–6.

83. Vakili R, Hashemian S. A Novel Mutation of Beta-ketothiolase Deficiency: The First Report from Iran and Review of Literature. Iran J Child Neurol. 2018;12:113–21.

84. Wakazono A, Fukao T, Yamaguchi S, Hori T, Orii T, Lambert M, et al. Molecular, biochemical, and clinical characterization of mitochondrial acetoacetyl-coenzyme A thiolase deficiency in two further patients. Hum Mutat. 1995;5:34–42.

85. Wen P, Chen Z, Wang G, Su Z, Zhang X, Tang G, et al. [Analysis of clinical phenotype and ACAT1 gene mutation in a family affected with beta-ketothiolase deficiency]. Zhonghua Yi Xue Yi Chuan Xue Za Zhi. 2016;33:286–91.

86. Wilcken B, Wiley V, Hammond J, Carpenter K. Screening newborns for inborn errors of metabolism by tandem mass spectrometry. N Engl J Med. 2003;348:2304–12.

87. Wojcik MH, Wierenga KJ, Rodan LH, Sahai I, Ferdinandusse S, Genetti CA, et al. Beta-Ketothiolase Deficiency Presenting with Metabolic Stroke After a Normal Newborn Screen in Two Individuals. JIMD Rep. 2018;39:45–54.

88. Yalçinkaya C, Apaydin H, Ozekmekçi S, Gibson KM. Delayed-onset dystonia associated with 3-oxothiolase deficiency. Mov Disord. 2001;16:372–5.

89. Yamaguchi S, Orii T, Sakura N, Miyazawa S, Hashimoto T. Defect in biosynthesis of mitochondrial acetoacetyl-coenzyme A thiolase in cultured fibroblasts from a boy with 3-ketothiolase deficiency. J Clin Invest. 1988;81:813–7.

90. Yang Y, Jiang SH, Liu S, Han XY, Wang Y, Wang LL, et al. Two Infants With Beta-Ketothiolase Deficiency Identified by Newborn Screening in China. Front Genet. 2019;10:451.

91. Yeste Fernández D, Castelló Girona F, Mora Graupera J, Riudor Taravila E, Arranz Amo J, Ribes Rubió A, et al. [Ketoacidotic coma in an infant as the form of onset of a mitochondrial 2-methylacetoacetyl-CoA thiolase deficiency]. An Esp Pediatr. 1996;44:620–2.

92. Zhang GX, Fukao T, Rolland M-O, Zabot M-T, Renom G, Touma E, et al. Mitochondrial acetoacetyl-CoA thiolase (T2) deficiency: T2-deficient patients with “mild” mutation(s) were previously misinterpreted as normal by the coupled assay with tiglyl-CoA. Pediatr Res. 2004;56:60–4.

93. Zhang G, Fukao T, Sakurai S, Yamada K, Michael Gibson K, Kondo N. Identification of Alu-mediated, large deletion-spanning exons 2-4 in a patient with mitochondrial acetoacetyl-CoA thiolase deficiency. Mol Genet Metab. 2006;89:222–6.
